# Solubility of CO_2_ in Aqueous Formic Acid Solutions and the Effect of NaCl Addition: A Molecular Simulation Study

**DOI:** 10.1021/acs.jpcc.2c05476

**Published:** 2022-11-04

**Authors:** Dominika O. Wasik, H. Mert Polat, Mahinder Ramdin, Othonas A. Moultos, Sofia Calero, Thijs J. H. Vlugt

**Affiliations:** † Materials Simulation and Modelling, Department of Applied Physics, Eindhoven University of Technology, Eindhoven5600MB, The Netherlands; ‡ Eindhoven Institute for Renewable Energy Systems, Eindhoven University of Technology, P.O. Box 513, Eindhoven5600 MB, The Netherlands; § Engineering Thermodynamics, Process & Energy Department, Faculty of Mechanical, Maritime and Materials Engineering, Delft University of Technology, Leeghwaterstraat 39, Delft2628CB, The Netherlands; ∥ CCUS and Acid Gas Entity, Liquefied Natural Gas Department, Exploration Production, TotalEnergies S.E., Paris92078, France; ⊥ CTP—Centre of Thermodynamics of Processes, Mines ParisTech, PSL University, 35 rue Saint Honoré, Fontainebleau77305, France

## Abstract

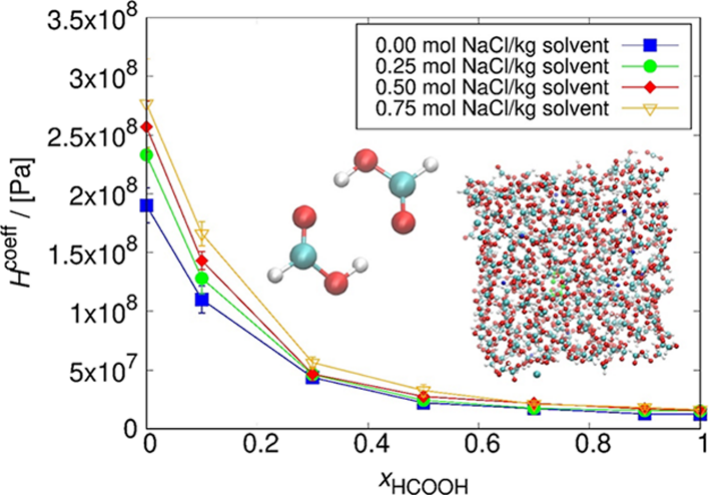

There is a growing interest in the development of routes to produce formic acid from CO_2_, such as the electrochemical reduction of CO_2_ to formic acid. The solubility of CO_2_ in the electrolyte influences the production rate of formic acid. Here, the dependence of the CO_2_ solubility in aqueous HCOOH solutions with electrolytes on the composition and the NaCl concentration was studied by Continuous Fractional Component Monte Carlo simulations at 298.15 K and 1 bar. The chemical potentials of CO_2_, H_2_O, and HCOOH were obtained directly from single simulations, enabling the calculation of Henry coefficients and subsequently considering salting in or salting out effects. As the force fields for HCOOH and H_2_O may not be compatible due to the presence of strong hydrogen bonds, the Gibbs–Duhem integration test was used to test this compatibility. The combination of the OPLS/AA force field with a new set of parameters, in combination with the SPC/E force field for water, was selected. It was found that the solubility of CO_2_ decreases with increasing NaCl concentration in the solution and increases with the increase of HCOOH concentration. This continues up to a certain concentration of HCOOH in the solution, after which the CO_2_ solubility is high and the NaCl concentration has no significant effect.

## 1 Introduction

The industrial revolution started an extensive use of fossil fuels, resulting in the release of alarming amounts of CO_2_ gas into the atmosphere.^1^ In the past years, the development of technologies for reducing CO_2_ emissions has been in the forefront of research. From an economical point of view, a promising way of decreasing CO_2_ emissions is the capture of CO_2_ at the source of production (e.g., at industrial sites) and the conversion to value-added products, for example, formic acid, methanol, propylene, and urea.^1−3^ In the chemical industry, formic acid (FA, HCOOH) is a primary product with a production capacity of 800,000 tons per year as of 2017.^4,5^ A wide range of products are synthesized from FA.^6−9^ It is, for example, used for the hydrothermal decarbonylation and decarboxylation in the water–gas-shift reaction,^6^ as a preservative (antibacterial cocktail in the prevention of potato spoilage^7^), as an antibacterial in livestock feed (control of luminous vibriosis disease in shrimp aquaculture^8^), and as a hydrogen storage material.^9^

Predominantly, formic acid is synthesized by methanol carbonylation resulting in a formate ester, which undergoes a hydrolysis process with an excess of water.^10−13^ Another method for producing formic acid is the electrochemical reduction of CO_2_ in an aqueous electrolyte solution.^14−17^ The electrochemical conversion of CO_2_ in a single step using H_2_O is less resource intensive than methanol carbonylation, uses an abundant resource (CO_2_), and avoids the production of intermediates. Electrochemical reduction of CO_2_ is typically performed in alkaline media to suppress the competing hydrogen evolution reaction (HER).^18^ The reduction of CO_2_ at the cathode results in formate (HCOO^–^) and hydroxide (OH^–^) ions^14^
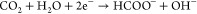
1

Depending on the pH, either formic acid or formate is produced. CO_2_ reduction is mostly performed in alkaline solutions,^14^ but this results in formate, which is not the desired product from a market perspective.^18^ To obtain FA, researchers are shifting to CO_2_ reduction in (slightly) acidic conditions using pH neutral electrolytes (such as NaCl), which become acidic upon CO_2_ dissolution.^18^ Due to possible salting-out effects, there is a trade-off between CO_2_ solubility and electric conductivity as a function of NaCl concentration.

Currently, electrocatalytic reduction of CO_2_ to HCOOH is not optimal due to the low solubility of CO_2_ in aqueous solutions.^19^ One possible solution is to use CO_2_ at high pressures.^16^ Another solution is to use a gas diffusion electrode, but in this case, fouling and/or undesired crystallization takes place.^20−22^ This is a problem for producing HCOOH as the low solubility of CO_2_ decreases the yield of the process. It is therefore crucial to investigate the possibilities of improving the solubility of CO_2_ in the electrolyte. Formic acid/water systems are difficult to study experimentally because FA is susceptible to decomposition at relatively low temperatures and exhibits complex behavior with mixtures.^23,24^ FA forms strong hydrogen bonds.^24−26^ There are two types of hydrogen bonds formed between FA monomers and water molecules: C–H···O and O–H···O.^25,27^

Due to the complex behavior of FA mixtures, force field-based molecular simulations are an alternative approach to obtain thermodynamic properties, in cases where there is a lack of experimental data. In this work, molecular simulations have been used to study the CO_2_ solubility in aqueous FA solutions and the effect of salting in/out. The modeling of hydrogen bonds is challenging in simulations because their presence leads to density and structural anomalies, such as a tetrahedral coordination in water.^28^ Due to the strong H-bonds of FA, systems of FA–water are difficult to model.^25,29−31^ To the best of our knowledge, the effect of salt addition on the CO_2_ solubility was investigated in pure water^32−35^ and not in aqueous FA solutions. The first attempt to make an economic evaluation of electrochemical reduction of CO_2_ with the use of potassium hydroxide and potassium sulfate (K_2_SO_4_) as electrolytes is described by Ramdin et al.^18^ The results of our work can be used for a better design of processes for the electrochemical reduction of CO_2_, in search of chemicals that enhance the CO_2_ solubility and thus the efficiency of the CO_2_ conversion without decreasing the ionic conductivity of an electrolyte solution. Here, we will show the salting out effect of the NaCl electrolyte on CO_2_ solubility and CO_2_ solubility dependence on HCOOH fraction in the solution.

This manuscript is organized as follows: in Section 2, we define the HCOOH model and force fields. In Section 3, we provide technical details of the molecular simulation methods. In Section 4, we specify detailed information on the simulations. In Section 5, we present and discuss the results. The selected HCOOH force field is validated by simulations of vapor–liquid equilibria (VLE) and vapor pressure as a function of temperature. The densities of HCOOH/H_2_O systems with different mole fractions of FA are compared to the experimental values. The dependence of the Henry coefficient of CO_2_ on the mole fraction of HCOOH and the NaCl concentration in the mixture is determined. The solubility of CO_2_ decreases with increasing NaCl concentration in the solution but increases with HCOOH concentration. Our findings are summarized in Section 6.

## 2 Force Field

**Figure 1:**
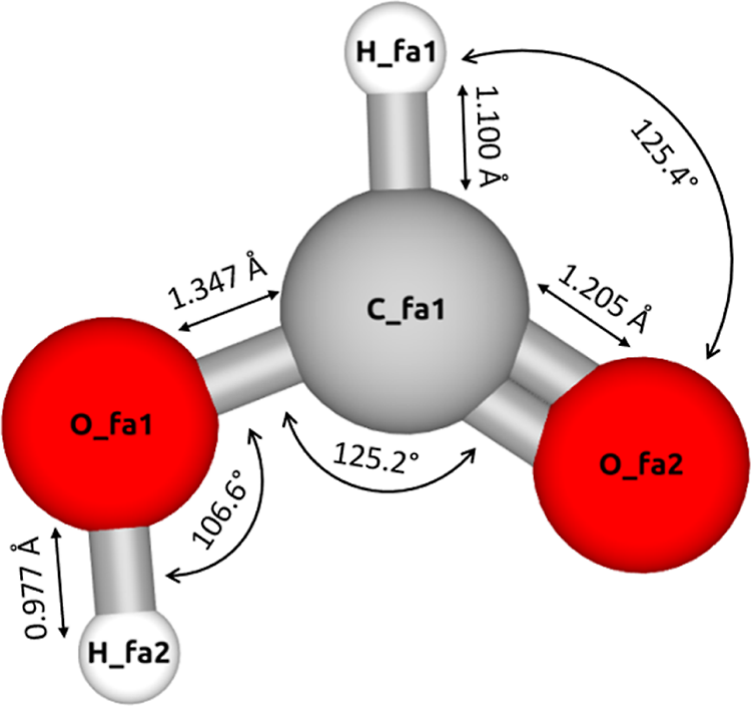
Schematic representation of the HCOOH model optimized using Gaussian09^37^ at the B3LYP/6-31G(d) level of theory. The visualization is created using iRASPA.^39^ The atom labels and the respective atomic positions are listed in Table S1 of the Supporting Information. The listed bond lengths are in Å.

The HCOOH molecule was constructed using the Avogadro molecule editor,^36^ and its geometry optimization was performed at the B3LYP/6-31G(d) level of theory using Gaussian09.^37^ The resulting geometry is shown in Figure 1. The model is rigid, and all interaction sites are at the atom positions (in sharp contrast to the anisotropic force field in the study by Schnabel et al.^38^).

Equilibration of systems containing both HCOOH and H_2_O is not straightforward due to the presence of strong hydrogen bonds.^40^ The compatibility of force fields for formic acid and water is unknown. For this reason, it is necessary to conduct a test verifying if the force field results in molecular simulations that are in thermodynamic equilibrium. Four force fields for HCOOH (FF-0, FF-1, FF-2, and FF-3) in combination with the SPC/E force field for water were studied here. In the literature, there are three variants of the OPLS/AA force field^41^ available for HCOOH, as well as a force field developed by Schnabel et al.^38^ The OPLS/AA force fields are here named FF-0, FF-1, and FF-2. They correspond to the so-called “Original”, P1 and P2 force fields from the study of Salas et al.,^41^ respectively. FF-3 is the force field by Schnabel et al.^38^ All the studied force fields use a functional form for intermolecular interactions based on Coulombic and Lennard-Jones (LJ) interactions
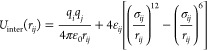
2where *r*_*ij*_ is the distance between atoms *i* and *j*, *q*_*i*_ is the partial charge of atom *i*, and ε_0_ is the permittivity of vacuum. ε_*ij*_ and σ_*ij*_ are the Lennard-Jones energy and size parameters, respectively.^41^ Interactions between unlike Lennard-Jones sites are defined by the Lorentz–Berthelot mixing rules.^42^ The interaction parameters for all the studied HCOOH force fields are shown in Tables S2–S5 of the Supporting Information, and references to the original publications of these force fields are provided. The interaction parameters of the HCOOH force field FF-2 selected for further simulations are in Table S4 of the Supporting Information, together with the SPC/E force field for water,^43^ the García-Sánchez et al. force field for CO_2_,^44^ and the Joung–Cheatham force field for NaCl,^45^ which were used in this work.

## 3 Methodology

The computation of excess chemical potentials (μ^ex^) enables to test the compatibility of force fields and subsequently the calculation of Henry coefficients for CO_2_ in aqueous solutions of HCOOH. The aim is to consider the salting in/salting out effects of NaCl addition to increase the electric conductivity and therefore the efficiency of the electrochemical reduction of CO_2_.

For all simulations, the Monte Carlo (MC) Software Brick-CFCMC^46,47^ was used. This is an open-source molecular simulation code for the calculation of phase and reaction equilibria using state-of-the-art force field-based MC simulations in different ensembles, such as the *NVT*, *NPT*, grand-canonical, reaction, and Gibbs ensemble.^47^ The Continuous Fractional Component (CFC) Monte Carlo^48,49^ method in the *NPT* ensemble can be considered as an expanded ensemble version of the conventional *NPT* ensemble, in which a fractional molecule is introduced to the system. The interactions of the fractional molecule are scaled by a parameter λ ∈ [0, 1]. There are no interactions of the fractional molecule with the surroundings when λ = 0, which means that the molecule is treated as an ideal gas molecule. For λ = 1, the fractional molecule has the same interactions as the other molecules not being a fractional molecule.^46,48^ The biasing of λ using a weight function [*W*(λ)] is necessary to prevent the system from getting stuck at certain values of λ.^48^ The weight function can be obtained via the Wang–Landau algorithm or an iterative scheme.^46^ The excess chemical potential can be calculated using two different routes.^46,47^ The first route is based on the probability distribution of the scaling factor [*p*(λ)] of the fractional molecule
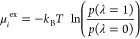
3where *k*_B_ is the Boltzmann constant, *T* is the temperature, and *p*(λ_*i*_ = 1) and *p*(λ_*i*_ = 0) are the probabilities of the scaling factor λ_*i*_ taking the value 1 and 0, respectively.^50,51^ A flat probability distribution of λ is ensured by adding a biasing weight function, which is easily achieved for small molecules (low uncertainty for μ^ex^). The second route is recommended in the case of large and/or strongly polar molecules and uses the computation of μ^ex^ via thermodynamic integration^47^
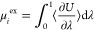
4

The application of thermodynamic integration eliminates the need for sampling the full λ-space with equal probability in a single simulation. The term ⟨∂*U*/∂λ⟩ is the ensemble average derivative of the potential energy with respect to the interaction scaling factor λ. Values for ⟨∂*U*/∂λ⟩ can be computed from several independent simulations at different fixed values of λ.^47,52^

The excess chemical potentials for HCOOH and H_2_O were calculated using the probability distribution of the scaling factor *p*(λ), as well as from thermodynamic integration. A series of *NPT* simulations of HCOOH/H_2_O systems were performed with mole fractions *x*_HCOOH_ = 0, 0.1, 0.3, 0.5, 0.7, 0.9, and 1. The compositions and average box volumes of all the seven systems simulated for the Gibbs–Duhem integration test are shown in Table S6 of the Supporting Information. The value of μ^ex^ computed from the probability distribution *p*(λ) was obtained directly from a single simulation of each system. Two fractional molecules (one for HCOOH and one for H_2_O) were introduced in those simulations. For the thermodynamic integration, simulations of each system had to be computed separately for a HCOOH fractional molecule and a H_2_O fractional molecule. In Brick-CFCMC, the value ⟨∂*U*/∂λ⟩ can only be computed for a single charge-neutral group of fractional molecules.^47^ A series of 99 simulations were performed at values of λ ranging from 0.01 to 0.99. The resulting values of ⟨∂*U*/∂λ⟩ as a function of λ were used in the thermodynamic integration (eq 4) to compute μ^ex^.

The computed values of μ^ex^ and the activity coefficient γ_*i*_ for component *i* depend on the composition of the system. The activity coefficient can be computed from^53^
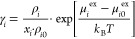
5where ρ_*i*_ and ρ_*i*0_ are the number densities of component *i* in the mixture and the reference number density of the pure solvent, respectively. μ_*i*_^ex^ is the excess chemical potential of component *i* in the mixture, and μ_*i*0_^ex^ is the excess chemical potential of *i* in the pure fluid *i*. The derivation of eq 5 is provided in the Supporting Information.

The Gibbs–Duhem integration test^54^ is a convenient tool to verify that the calculated activity coefficients of a system correspond to a system at equilibrium. Four studied HCOOH force fields^38,41^ in combination with the SPC/E force field for water were checked for thermodynamic consistency using the Gibbs–Duhem integration test^54^
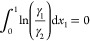
6where γ_1_ and γ_2_ are the activity coefficients of component 1 and component 2, respectively, and *x*_1_ is a mole fraction of component 1. The trapezoidal rule^55^ was used for approximating the definite integral
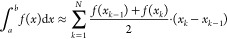
7where *N* is the number of the subintervals. The uncertainties of the Gibbs–Duhem integrals were calculated using error propagation. The uncertainty for the *k*th subinterval of the integral is calculated by
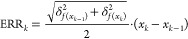
8where  is an uncertainty of  computed as the standard deviation from five independent simulations and *x*_*k*_ is a mole fraction of component 1, considered in the *k*th subinterval. The uncertainty of the Gibbs–Duhem integral is calculated using all subintervals by
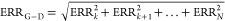
9

The densities of simulated HCOOH/H_2_O binary mixtures with different HCOOH mole fractions were compared with the experimental data^43^ at temperatures 288.15, 298, and 303.15 K. The VLE curve for pure HCOOH was simulated using the Gibbs ensemble at a constant total volume, combined with the CFC method.^46^ In this ensemble, there are two simulation boxes that can exchange molecules and volume. The phase equilibrium densities were reproduced for a temperature range from 335 to 560 K and compared to the experimental data.^38^ At the start of the simulation, the total number of molecules in each simulation was equal to 400. Both simulation boxes were identical in terms of number of molecules and volume. The critical values of temperature and density were determined using the Schröer–Pottlacher approach.^56^ The densities of the gas phase from the Gibbs ensemble simulations were used to compute saturated vapor pressures. For the temperature range 335–560 K, a series of *NPT* simulations for the gas phase were performed as a function of pressure. From these simulations, HCOOH vapor pressures were calculated using interpolation of the pressure as a function of density. Additionally, vapor pressures of HCOOH were calculated by assuming an ideal gas phase^57^
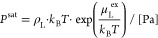
10where ρ_L_ and μ_L_^ex^ are the number density and excess chemical potential of the liquid phase from Gibbs ensemble simulations, respectively. This approximation is investigated in the Supporting Information by the calculation of HCOOH dimer and monomer partial vapor pressures.

The solubilities of CO_2_ in aqueous HCOOH solutions were determined from the Henry coefficients, which are calculated by^58^
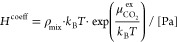
11where ρ_mix_ is a number density of the mixture and  is the excess chemical potential of CO_2_ at infinite dilution. To investigate the dependence of CO_2_ solubility on the salt content, *NPT* simulations of systems containing HCOOH/H_2_O/CO_2_/NaCl were performed. To characterize the HCOOH content, HCOOH pseudo-mole fractions were used that are defined by
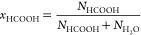
12

For each HCOOH pseudo-mole fraction *x*_HCOOH_ = 0, 0.1, 0.3, 0.5, 0.7, 0.9, and 1, four concentrations of NaCl have been studied: 0, 0.25, 0.5, and 0.75 mol NaCl per kilogram of solvent (HCOOH + H_2_O). The exact number of molecules used in each system is shown in Table S7 of the Supporting Information. To enable the calculation of the excess chemical potential of CO_2_, a fractional molecule of CO_2_ is added to all systems.

As this is our first estimate for the HCOOH/H_2_O/NaCl mixture, the ideal gas behavior was assumed. The computations are performed in the limit where the pressure approaches zero and hence the volume tends to infinity. In this limit, the side of monomers is favored by the reaction equilibrium in the gas phase. The increase of the volume leads to the decrease of the density. To counteract this, the number of monomers increases in the system, resulting in the increase of the density. The appearance of monomers only allows us to approximate the ideal gas behavior. The vapor pressures of H_2_O and HCOOH were calculated by^59^
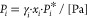
13where γ_*i*_ is an activity coefficient of component *i*, *x*_*i*_ is a mole fraction of component *i*, *P*_*i*_ is the actual partial vapor pressure, and *P*_i_^*^ is the vapor pressure of the pure solvent at the same temperature. The values of *P*_i_^*^ for HCOOH and H_2_O used in eq 13 were computed at 298.15 K using Gibbs ensemble simulations (at constant total volume). The computed values were equal to the experimental data at 298 K^60,61^ within the error bars. The values of *P*_i_^*^ for HCOOH and H_2_O used in eq 13 were 5678.2^60^ and 3169.0 Pa,^61^ respectively. The total pressure of the system was calculated according to the additivity of the partial pressures
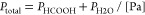
14where *P*_HCOOH_ and *P*_H_2_O_ are the partial pressures of HCOOH and H_2_O, respectively. The results were compared with the experimentally measured total pressures of the HCOOH/H_2_O mixture at 291.15^62^ and 303.15 K^63,64^ without NaCl added. The HCOOH vapor mole fraction was calculated by
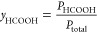
15

The azeotropic behavior of the simulated HCOOH/H_2_O system without NaCl addition was analyzed using *P*(*x*,*y*) and *y*–*x* diagrams. The results were compared with the NRTL–HOC method^65,66^ using parameters from ref (18).

The effect of salt on the solubility of CO_2_ can be described by engineering models, for example, the study by Weisenberger and Schumpe.^67^ The solubility of CO_2_ was compared with an engineering model for estimation of gas solubilities in salt solutions.^67^ The Sechenov constant *K* was computed for each HCOOH pseudo-mole fraction from the slope of linear function, according to
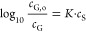
16where *c*_G,o_ and *c*_G_ are the gas solubilities in pure water and in the salt solution, respectively, and *c*_S_ is the molar concentration of the salt. The solubility is related to Henry coefficients computed in the simulations of HCOOH/H_2_O/CO_2_/NaCl mixtures and expressed as *H*^coeff-1^. The solubility of gases in the solvent is inversely proportional to the Henry coefficient. The obtained values for *K* were compared with the Sechenov constant for the CO_2_/NaCl system calculated based on engineering model parameters.^67^

## 4 Simulation Details

In all simulations, the cutoff radius for intermolecular interactions is set to 10 Å. Interactions are truncated with the analytic tail corrections applied. Periodic boundary conditions are exerted in all three directions. The Ewald summation method^68^ is used for calculating electrostatic interactions. The number of *k*-vectors in each direction *K*_max_ equals 8, and the damping parameter α equals 0.32 Å^–1^. The parameters for the Ewald summation correspond to a relative precision of 10^–6^. One single MC cycle consists of *N* MC trial moves, where *N* is the total number of molecules at the start of the simulation. Each simulation was carried out with 200,000 equilibration cycles. In the production phase, 400,000 to 500,000 MC cycles were performed, depending on the energy equilibration of individual systems and reaching flat probability distribution of the observed value of λ. The probabilities of selecting trial moves were 30% translations, 30% rotations, 1% volume changes, 15% λ changes, and 30% CFC hybrid moves that combine the swap and identity changes.^46^ To calculate the standard deviations of the computed values, all sets of simulations were performed five times starting from independent configurations and using different random number seeds. The algorithms provided in Brick-CFCMC^46,47^ are used to generate random initial configurations. The dissociation of formic acid in water was neglected in the simulations due to the low p*K*_a_ value of formic acid (3.745).^69^ As a rough guide, for an ideal solution model, the equilibrium reaction extent for the dissociation reaction (H^+^ and HCOO^–^) would be equal to only 0.026. We also neglected the soluble^70^ sodium formate since it forms at very low concentrations by reaction between the formate product of CO_2_ reduction and the cell electrolyte.

## 5 Results and Discussion

**Figure 2:**
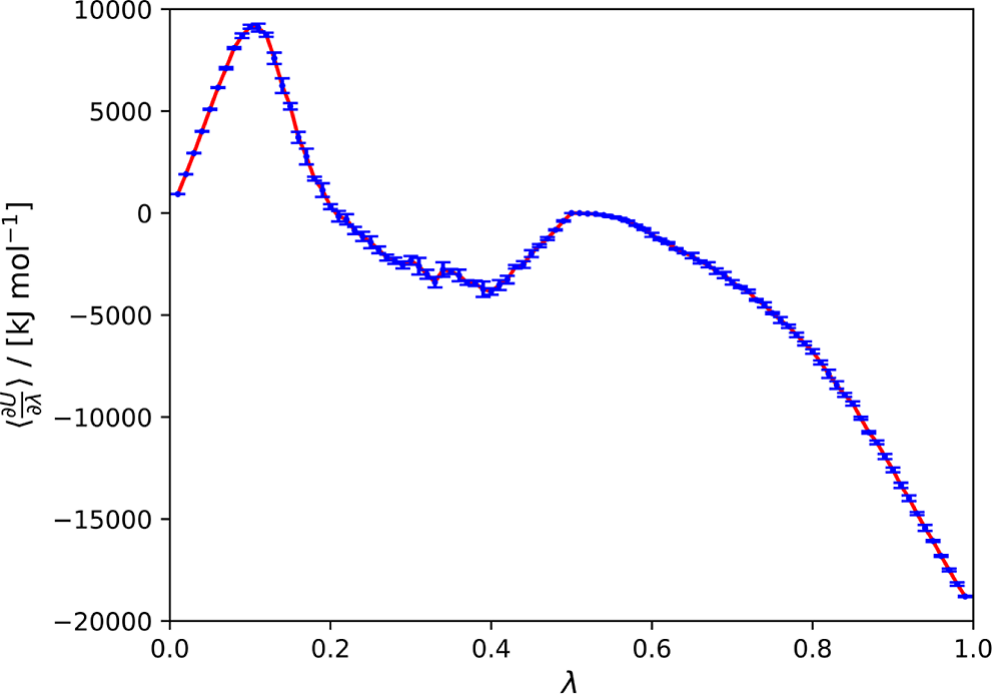
⟨∂*U*/∂λ⟩ as a function of λ for a HCOOH/H_2_O system containing a fractional molecule of HCOOH. *x*_HCOOH_ = 0.1, *T* = 298 K, and *P* = 1 bar. The values of ⟨∂*U*/∂λ⟩ were obtained from 99 independent simulations at different fixed values of λ. The blue points represent the values of ⟨∂*U*/∂λ⟩, and the red line is a fitted spline. The error bars of ⟨∂*U*/∂λ⟩ were computed as the standard deviation from five independent simulations. The integration of the fitted spline resulted in μ_ex_ of HCOOH equals to −24.5 ± 0.1 kJ mol^–1^.

We first validated for which combinations of the SPC/E–HCOOH force fields the Gibbs–Duhem integration is passed within the error bars. In Table S8 of the Supporting Information, the excess chemical potentials obtained from the probability distribution of λ and thermodynamic integration are compared. It turns out that both routes for obtaining μ^ex^ (i.e., from the probability distribution of λ and thermodynamic integration) lead to nearly identical results. The conceptually simpler route of using the probability distribution of λ turned out to be sufficiently accurate. The relative difference between μ^ex^ computed from the probability distribution of λ and μ^ex^ from thermodynamic integration is below 0.5%. The average uncertainties of μ^ex^ are 0.31 and 0.13 kJ mol^–1^ in the case of probability distribution of λ and thermodynamic integration, respectively. A typical example of ⟨∂*U*/∂λ⟩ as a function of λ is shown in Figure 2.

The Gibbs–Duhem integration test was performed based on the values of μ_ex_ of HCOOH and H_2_O computed from the probability distribution of λ. The values of  for four studied force fields as a function of HCOOH mole fraction are shown in Figure S1 of the Supporting Information. Table 1 shows the results of this integration. For further studies, we selected the FF-2 force field,^41^ as the OPLS/AA force field is a well-established, broadly applicable, and extensively developed force field for organic molecules.^71^ The HCOOH force field FF-2 is used for all simulations in the remainder of this paper. The activity coefficients and densities for HCOOH and H_2_O obtained using all the studied force fields are listed in Tables S9 and S10 of the Supporting Information.

**Table 1:** Gibbs–Duhem Integration Test Results for the Four Studied HCOOH/H_2_O Force Fields^a^

force field	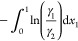
FF-0	0.08_0.10_
FF-1	0.01_0.13_
FF-2	–0.03_0.11_
FF-3	0.02_0.16_

a All the studied force fields resulted in the Gibbs–Duhem integral equals to zero within the error bars. The subscripts show uncertainties computed using error propagation rules.

**Figure 3:**
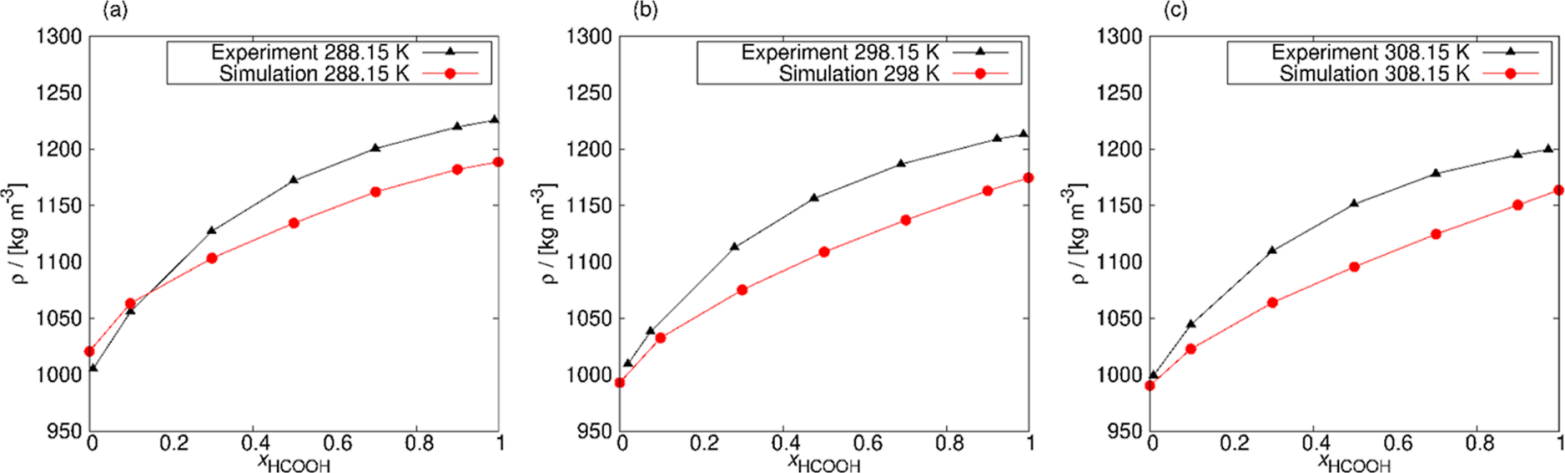
Densities of HCOOH/H_2_O systems as a function of the mole fraction of HCOOH compared to the experimental values^72^ at (a) 288.15 K, (b) 298 K, and (c) 308.15 K and 1 bar. Calculated values of density differ from the experimental values by 0.71–3.19% for 288.15 K, 0.52–4.16% for 298 K, and 0.90–4.82% for 308.15 K. The uncertainties were computed as the standard deviation from five independent simulations. The error bars are smaller than the size of the symbols.

For validation of the FF-2 HCOOH/H_2_O SPC/E force fields, the simulated HCOOH/H_2_O densities were compared with the experimental densities found in the literature^72^ in Figure 3. Three temperatures were considered: 288.15, 298, and 308.15 K. The values of the calculated densities differ from the experimental ones by 0.52–4.82%. The order of magnitude for uncertainty values is ca. 1 g cm^–3^.

**Figure 4:**
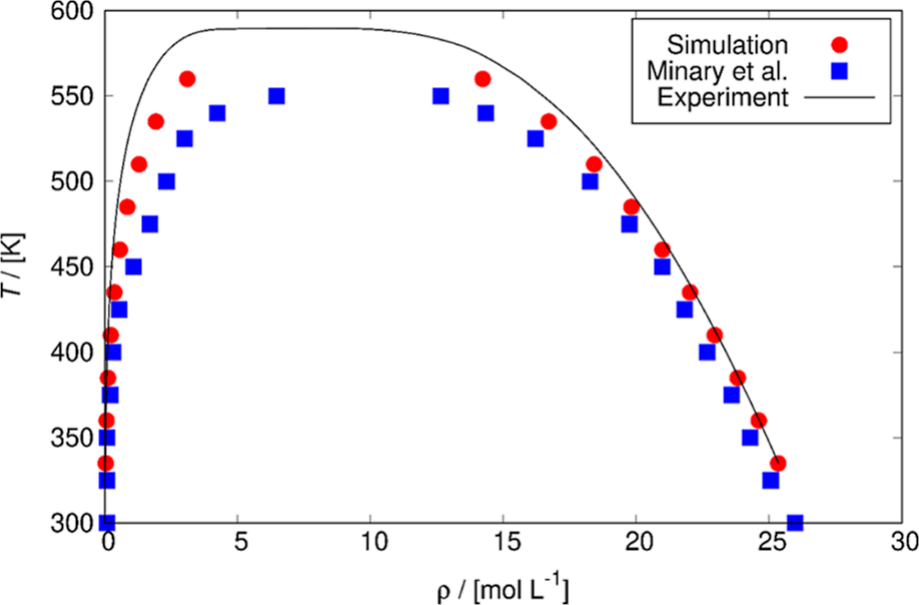
Vapor–liquid equilibrium curve of pure HCOOH. The coexistence densities were reproduced for a temperature range from 335 to 560 K and compared with experimental data^38^ and simulation results of Mináry et al.^73^ The uncertainties were computed as the standard deviation from five independent simulations. The error bars are smaller than the size of the symbols. The average uncertainty of the computed densities is 0.021 mol L^–1^. The simulated VLE curve fits well with the experimental values up to 510 K, after which values start to diverge. The critical values of temperature and density were determined (experimental values in parentheses): *T*_c_ = 610.19 (588.00)^38^ K, ρ_c_ = 7.39 (8.00)^38^ mol L^–1^, leading to the differences relative to the experimental values, respectively, 3.8 and 7.6%.

The FF-2 force field was further validated for a pure HCOOH system. The VLE phase diagram was simulated for a temperature range from 335 to 560 K and compared with experimental values^38^ and simulation results of Mináry et al.^73^ (see Figure 4). The computed VLE curve fits well with the experimental values up to 510 K. At higher temperatures, the line starts to diverge from the experimental values. The average uncertainty of computed densities is 0.021 mol L^–1^. The VLE curve obtained using the FF-2 force field is less deflected from the experimental data than the simulation results of Mináry et al.^73^ using the HCOOH model of Jedlovszky and Turi.^25^

**Figure 5:**
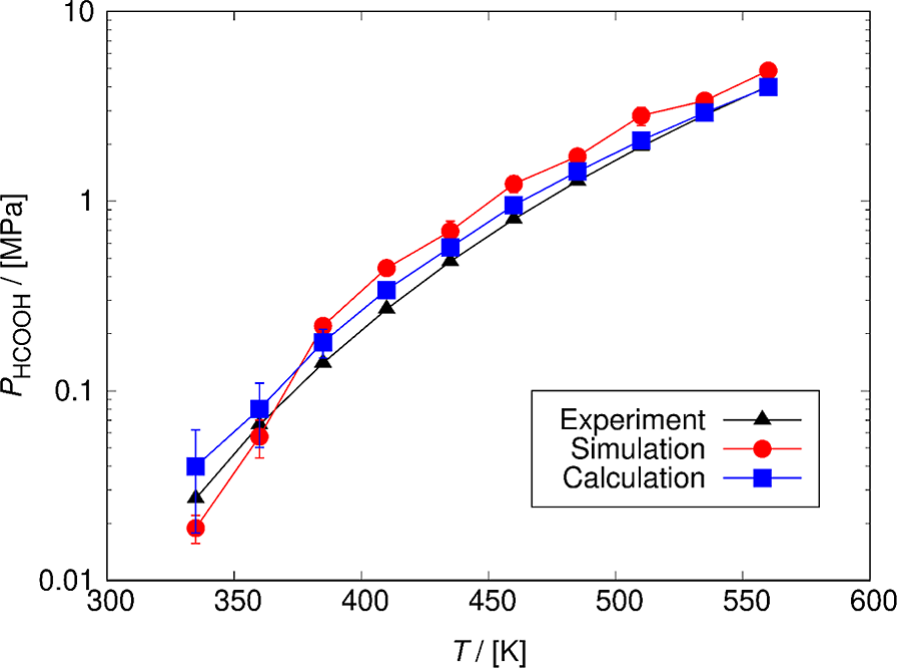
Comparison of the saturated vapor pressures of HCOOH computed from a series of *NPT* simulations of vapor phase and calculated using the liquid-phase properties from Gibbs ensemble simulations with experimental values^38^ as a function of temperature. In the case of the vapor-phase simulations, the differences between simulations and experiments vary by 13.26–64.74% due to the small number of molecules used in the simulation. The saturated vapor pressures calculated using eq 10 differ from the experimental values by 37.84–1.19%, whereby with increasing vapor pressure, the difference decreases. The values of the HCOOH vapor pressures are shown in Table S11. The uncertainties are computed as the standard deviation obtained from five independent Gibbs ensemble simulations. In the case of the vapor pressures of pure HCOOH computed from the series of NPT simulations (*P*_HCOOH,sim_), the average uncertainty equals to 0.1 MPa. The average uncertainty of the values calculated by eq 10 is equal to 0.02 MPa.

The saturated vapor pressure of pure FA was computed from a series of *NPT* simulations of vapor phase and calculated using the liquid-phase properties from Gibbs ensemble simulations using eq 10. The results were compared with experimental data^38^ as a function of temperature, as shown in Figure 5. In the case of vapor-phase simulations, the vapor pressures of pure FA differ from the experimental ones by 13.26–64.74%. The average deviation equals to 29.66%. The uncertainties are close to zero due to the small number of molecules used in the simulation of the vapor box. The average uncertainty equals to 0.1 MPa. The configurations of computed systems were visualized, and the presence of dimers was confirmed in the gas phase of our simulations (see Figure S2 in the Supporting Information). The formation of dimers in FA is experimentally proven and leads to a non-ideal gas behavior.^24,31^ The values of saturated vapor pressures calculated using eq 10 differ from the experimental data by 37.84–1.19%. The difference decreases with increasing vapor pressure. The average uncertainty of the calculated pressures is equal to 0.02 MPa.

**Figure 6:**
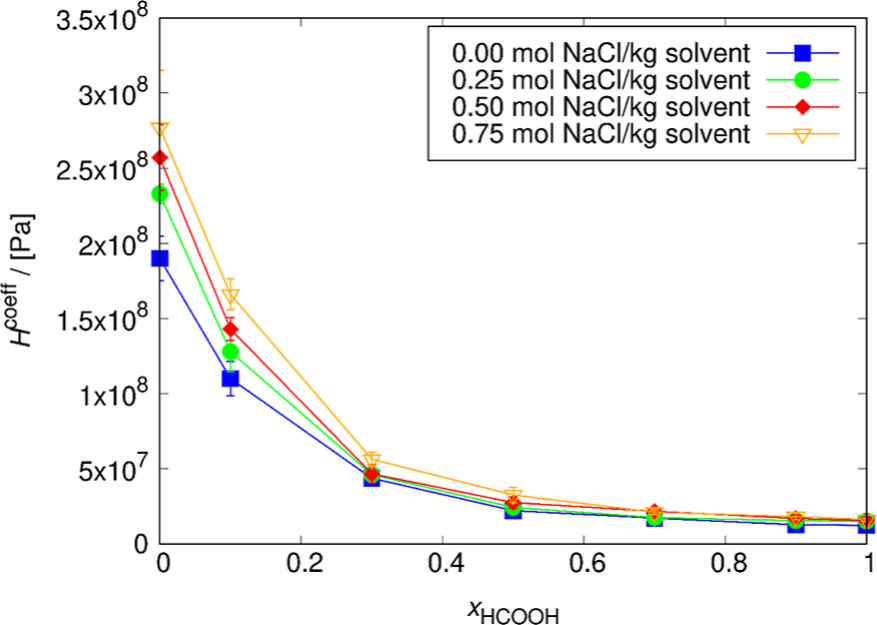
Henry coefficients of CO_2_ computed for several NaCl concentrations as a function of the pseudo-mole fraction of HCOOH at 298.15 K. The lines connecting the symbols are used to guide the eye. The solubility of CO_2_ decreases with the increase of the NaCl concentration in the solution but increases with the HCOOH concentration. The uncertainties were computed as the standard deviation from five independent simulations. The uncertainty decreases with the increase of HCOOH concentration. For *x*_HCOOH_ = 0, the order of magnitude of the error bars is 1 × 10^7^ Pa; for *x*_HCOOH_ = 0.3, this drops to 1 × 10^6^. Compared to the experimental Henry coefficient for CO_2_ in water, which is 1.62 × 10^8^ Pa at 298.15 K,^74^ the simulated value differs by 16.9% and equals to 1.90 × 10^8^ ± 1 × 10^7^ Pa.

**Figure 7:**
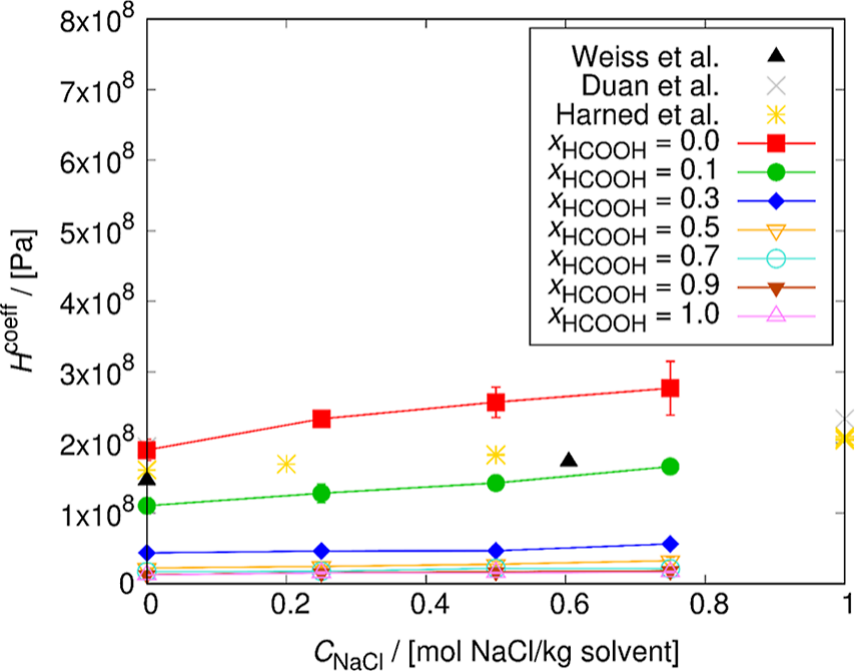
Henry coefficients of CO_2_ computed for different HCOOH pseudo-mole fractions as a function of the NaCl concentration in the solution at 298.15 K. The lines connecting the symbols are used to guide the eye. The solubility of CO_2_ decreases with the increase of the NaCl concentration in the solution but increases with the HCOOH concentration. The uncertainties were computed as the standard deviation from five independent simulations. The uncertainty decreases with the increase of HCOOH concentration. The experimental Henry coefficients of CO_2_ for the H_2_O/NaCl system at 293.8 K from a study of Weiss et al.^75^ are displayed as black points. The Henry coefficients calculated from experimental solubilities at 298.15 K from a study of Harned and Davis^76^ are displayed as yellow points. The calculated Henry coefficients from a model of Duan and Sun^32^ at 303.15 K are displayed as gray points.

The dependence of the Henry coefficient of CO_2_ on the mole fraction of HCOOH and NaCl concentration in the mixture was computed. The results are presented in Figures 6 and 7.

In Figure 7, the literature values for CO_2_ solubility in the H_2_O/NaCl system are shown for comparison. These are as follows: (1) experimental Henry coefficients of CO_2_ for the H_2_O/NaCl system at 293.8 K from a study of Weiss et al.,^75^ (2) Henry coefficients calculated from experimental solubilities at 298.15 K from a study of Harned and Davis,^76^ and (3) Henry coefficients calculated from experimental solubilities at 303.15 K from a model of Duan and Sun.^32^ From the comparison with the literature, it is shown that the Henry coefficient of CO_2_ computed in this study is in the correct range. The average deviation from the experimental values at 298.15 K^76^ is 31%. The reason of deviations from the experimental data is the non-ideal gas behavior.

**Figure 8:**
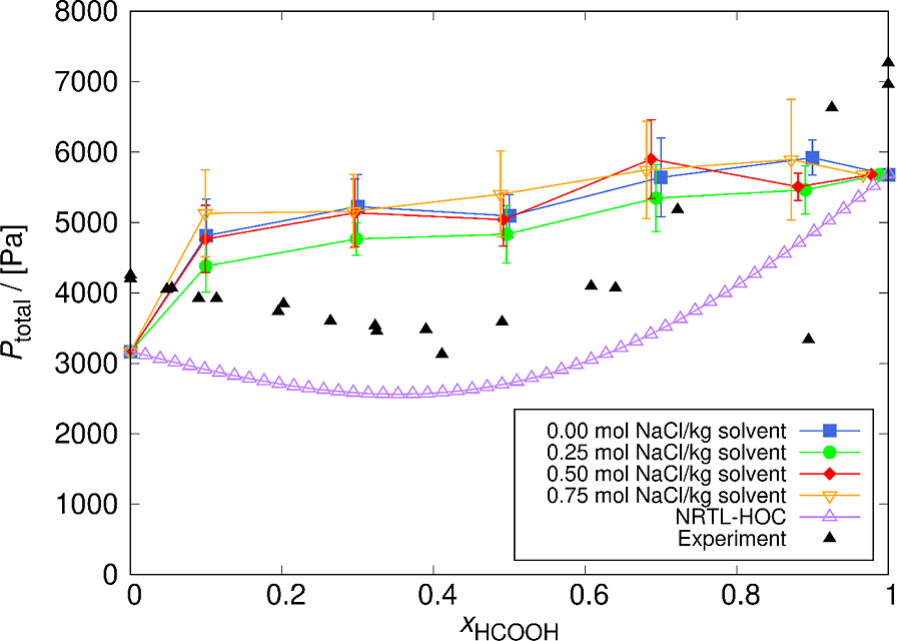
Total vapor pressure (*P*_H_2_O_ + *P*_HCOOH_) for systems of certain NaCl concentrations as a function of the HCOOH mole fraction at 298.15 K. The lines connecting the symbols are used to guide the eye. For comparison, vapor pressures of the NRTL-HOC model at 298.15 K^18^ and experimental vapor pressures at 291.15^62^ and 303.15 K^63,64^ for a system without NaCl are also shown. The uncertainties were computed as the standard deviation from five independent simulations.

In Figure 8, vapor pressures for systems with different NaCl concentrations are shown as a function of the pseudo-mole fraction of HCOOH. Due to the lack of experimental data at 298.15 K, the experimental vapor pressures for a system without added NaCl are shown for comparison at 291.15^62^ and 303.15 K.^63,64^ The difference between temperature used in our study and temperature of the experimental vapor pressures for a system without NaCl is very small, and the resulting vapor pressures are comparable.

The computed vapor pressures are in the correct range but show a relatively high deviation from experimental values. The simulations result in a low-boiling azeotrope in sharp contrast to the NRTL-HOC computations^18^ that indicate high-boiling azeotrope behavior of the HCOOH/H_2_O system (see Figure S3 of the Supporting Information). Our first estimate of the HCOOH/H_2_O/NaCl model did not describe the azeotrope accurately due to the non-ideal gas behavior caused by dimer formation, which was confirmed by visually inspecting characteristic configurations of the simulations as shown in Figure S4 of the Supporting Information. Achieving the same magnitude of computed and experimentally measured vapor pressures is considered as sufficient for our HCOOH/H_2_O/NaCl model without any adjustments. Additionally, the calculation of HCOOH dimer and monomer partial vapor pressures is presented in Table S12 of the Supporting Information. The calculated dimer partial vapor pressures are found to be higher than the monomer partial vapor pressures, confirming that the non-ideal dimer formation behavior is the reason why our model does not reproduce vapor pressures and azeotropic behavior more precisely than the order of magnitude. The Sechenov constants for each studied pseudo-mole fraction of HCOOH are listed in Table 2. The value of *K* calculated using model parameters^67^ for the CO_2_/NaCl system is 0.1117 m^3^ kmol^–1^. The computed *K* for different pseudo-mole fractions of HCOOH shows a scattering but is of the same order of magnitude. The experimental solubility of NaCl in pure formic acid is 0.89 mol per kg of solvent at 298.15 K.^77^ NaCl is still soluble for all the studied concentrations in the simulated systems.

**Table 2:** Sechenov Constants for Each Studied Pseudo-mole Fraction of HCOOH^a^

*x*_HCOOH_	*K*/[m^3^ kmol–^1^]
0	0.2_0.1_
0.1	0.23_0.08_
0.3	0.11_0.04_
0.5	0.19_0.06_
0.7	0.13_0.09_
0.9	0.19_0.06_
1	0.16_0.05_

a The value of *K* calculated using model parameters^67^ for the CO_2_/NaCl system is 0.1117 m^3^ kmol^–1^. The error bars were computed as the standard deviation from five independent simulations. The computed values of *K* for different pseudo-mole fractions of HCOOH are in the same order of magnitude.

The solubility of CO_2_ increases with the HCOOH concentration (decrease in Henry constant), leading to the highest solubility for pure formic acid and the lowest in the case of pure water. The CO_2_ solubility decreases with the increase of NaCl concentration in the solution. This trend continues up to *x*_HCOOH_ = 0.3 in the solution, after which the concentration of NaCl has no significant effect on CO_2_ solubility. This effect is described as salting out, where an increase in the ionic strength of a solution decreases the solubility of a solute. The more notable decrease in the CO_2_ solubility for systems consisting predominantly of water can be explained by the fact that when salt ions such as NaCl are added to water, water molecules are bound to “solvates”, leaving fewer water molecules for CO_2_ to adhere to. Upon salt addition, weak attractions of CO_2_ molecules to water are decreased, and dissolved CO_2_ is displaced from polar water. Studies on the hydration of ions and the interaction of ions with water molecules have shown that, at a high density, smaller ions tend to bind the molecules of water more effectively, while larger ions with a low charge density bind the water molecules weakly.^78,79^ Similar results for CO_2_ solubility in water in the presence of salt ions are presented in the literature.^32−35^ Salting out effects were shown in a study by Liu et al.,^33^ where systems consisting of distilled water and various concentrations of NaCl, MgCl_2_, CaCl_2_, and MgCl_2_ + CaCl_2_ were studied. The solubilities of CO_2_ were measured in pure distilled water and salinities of 1000, 10,000, and 15,000 ppm at 298 K. In all cases, a decrease in CO_2_ solubility was observed as salinity increases. In a study by Koschel,^34^ the effect of NaCl concentrations on CO_2_ solubility in water was studied at 323.1 K and 2–20 MPa and at 373.1 K and 5–20 MPa. For both temperatures, salting out was observed, in line with the findings by Liu et al.^35^ on the effects of NaCl + KCl + CaCl_2_ at various temperatures.

An interesting research topic is to investigate if other chemicals may enhance the CO_2_ solubility. Salting-in effects are possible by using salts such as NaClO_4_.^80^ In the aqueous NaClO_4_ solutions, mutual affinity occurs between the ClO^4–^ anion and CO_2_.^81^ This results in an increase in the solubility of CO_2_. For most salting-out salts, the salting effect is possible to predict based on the viscosity B-coefficients^82^ that describe the change of water mobility induced by salts. The salting-in effect is more specific and complex than the salting-out. It cannot be predicted by the viscosity B-coefficients of salts. In the most of cases, there is a correlation between salting out Sechenov constant and viscosity B-coefficient.^80^ The structure maker ions (positive value of viscosity B-coefficients) strengthen the water–water hydrogen bond network and reduce the entropy of water. The solubility of the non-polar solutes decreases. The salting-in NaClO_4_ is an exception. It has a positive value of the viscosity B-coefficient (0.012^82^), whose inconsistency is not well understood due to the lack of knowledge on the underlying molecular mechanism.^80^

## 6 Conclusions

We present a study on the solubility of CO_2_ in aqueous solutions of HCOOH and the effect of adding NaCl to the system. All the studied HCOOH/SPC/E force fields resulted in the Gibbs–Duhem integral equals to zero within the error bars, even though they were not parameterized explicitly for mixtures with water. To investigate the compatibility of the other HCOOH force fields with the SPC/E force field, more research is required. The CFC Monte Carlo method turns out to be able to compute excess chemical potential with sufficient accuracy. The routes used to compute the excess chemical potentials of HCOOH and H_2_O, that is, (1) from the probability distribution of the scaling factor and (2) thermodynamic integration, resulted in excess chemical potentials that differ by approximately less than 0.5%. The method using *p*(λ) is precise enough to reproduce μ^ex^. Validation of the selected force field by comparing the density, vapor–liquid equilibrium, and vapor pressure with the experimental values showed a high accuracy of the model. Based on the most compatible model FF-2 HCOOH/H_2_O SPC/E, we observe the salting out effect of NaCl on CO_2_ solubility, which should be considered in further research in the field of CO_2_ reduction. From an economical point of view, the salting out effect is unprofitable because it reduces the amount of product obtained. It is also disadvantageous from an ecological point of view, as less CO_2_ would be reduced and removed from the environment. It would be interesting to investigate if other chemicals may enhance the CO_2_ solubility and hence the efficiency of the CO_2_ conversion without decreasing the ionic conductivity of an electrolyte solution. An important observation is that CO_2_ solubility increases (Henry coefficient decreases) with HCOOH fraction in the solution. HCOOH production may be a better alternative to elevating CO_2_ pressure to modify solubility.

## Supporting Information Available

Derivation of the equation for computing activity coefficients, details on the calculation of HCOOH monomer and dimer partial vapor pressures, information about the HCOOH models, force field parameters used in the simulations, compositions and average box lengths of all the studied systems, values of excess chemical potentials for HCOOH and H_2_O obtained from the probability distribution of the scaling factor *p*(λ) as well as thermodynamic integration, values of activity coefficients and densities for HCOOH and H_2_O, Gibbs–Duhem integration test for four studied HCOOH/H_2_O force fields as a function of HCOOH mole fraction, values of the simulated HCOOH vapor pressures, HCOOH dimer partial vapor pressures, and HCOOH monomer partial vapor pressures (PDF)
