# Supplementary material for: Solubility of CO2 in Aqueous Formic Acid Solutions and the Effect of NaCl Addition: A Molecular Simulation Study
Source: J Phys Chem C Nanomater Interfaces. 2022 Nov 4;126(45):19424–34. doi: 10.1021/acs.jpcc.2c05476 (PMC9677493; doi:10.1021/acs.jpcc.2c05476)
Supplement: Supplementary file 1 — jp2c05476_si_001.pdf [file jp2c05476_si_001.pdf]

Supporting Information for:

Solubility of CO_2 in Aqueous Formic Acid Solutions and the Effect of NaCl Addition: A Molecular Simulation Study

Dominika O. Wasik,[§⊥] H. Mert Polat,[‡†¶] Mahinder Ramdin,[‡] Othonas A. Moultos,[‡] Sofia Calero,[§⊥] Thijs J. H. Vlugt,[‡]

§Materials Simulation and Modelling, Department of Applied Physics, Eindhoven University of Technology, 5600MB Eindhoven, The Netherlands

⊥Eindhoven Institute for Renewable Energy Systems, Eindhoven University of Technology, PO Box 513, Eindhoven 5600 MB, The Netherlands

‡Engineering Thermodynamics, Process & Energy Department, Faculty of Mechanical, Maritime and Materials Engineering, Delft University of Technology, Leeghwaterstraat 39, Delft 2628CB, The Netherlands

†CCUS and Acid Gas Entity, Liquefied Natural Gas Department, Exploration Production, TotalEnergies S.E., 92078 Paris, France

¶CTP - Centre of Thermodynamics of Processes, Mines ParisTech, PSL University, 35 rue Saint Honoré, 77305 Fontainebleau, France

E-mail: t.j.h.vlugt@tudelft.nl

1 ACTIVITY COEFFICIENTS

The chemical potential of component *i* in a mixture with respect to the ideal gas reference state is described by[1,2]:

$$\mu_i = \mu_i^{\circ} + RT \ln\frac{\langle\rho_i\rangle}{\rho_0} + \mu_i^{\text{ex}} = \mu_i^{\circ} + RT \ln\frac{\langle\rho_i\rangle}{\rho_0} - RT \ln\frac{p(\lambda_i=1)}{p(\lambda_i=0)} \qquad \text{(S1)}$$

where μ_i° is the reference state of the chemical potential of component *i*, which depends on temperature but not on the pressure, $\langle\rho_i\rangle$ is the average number density of *i*, ρ_0 is the reference number density of the pure solvent, μ_i^{ex} is the excess chemical potential of *i*, $p(\lambda_i = 1)$ and $p(\lambda_i = 0)$ are the probabilities of the scaling factor λ_i taking the value 1 and 0, respectively. The chemical potential of component *i* in a mixture for a liquid-based reference state is expressed by[1]:

$$\mu_i = \mu_i^{*} + k_{\text{B}}T \ln(\gamma_i x_i) \qquad \text{(S2)}$$

where γ_i is the activity coefficients of component *i* and x_i is a mole fraction of component *i*. Combining Equations (S1) and (S2), the reference chemical potential μ_i^{*} is obtained for a pure component:

$$\mu_i^{*} = \mu_i^{\circ} + k_{\text{B}}T \ln\frac{\langle\rho_i\rangle}{\rho_0} + \mu_{0i}^{\text{ex}} \qquad \text{(S3)}$$

where μ_i^{*} is the reference state of the chemical potential of component *i*, which depends on temperature and pressure, μ_{0i}^{ex} is the excess chemical potential of *i* with respect to the ideal gas. Combining Equations (S2) and (S3) and neglecting the pressure-dependence of μ_i^{*}, the chemical potential of component *i* is:

$$\mu_i = \mu_i^{\circ} + k_{\text{B}}T \ln\frac{\langle\rho_i\rangle}{\rho_0} + \mu_{0i}^{\text{ex}} + k_{\text{B}}T \ln(\gamma_i x_i) \qquad \text{(S4)}$$

The activity coefficient is obtained by combining Equations (S1) and (S4):

$$k_{\text{B}}T \ln\frac{\langle\rho_i\rangle}{\langle\rho_{0i}\rangle} + \mu_i^{\text{ex}} - \mu_{0i}^{\text{ex}} = k_{\text{B}}T \ln(\gamma_i x_i) \qquad \text{(S5)}$$

$$\gamma_i = \frac{\langle\rho_i\rangle}{x_i \cdot \langle\rho_{i0}\rangle} \cdot \exp\left[\frac{\mu_i^{\text{ex}} - \mu_{i0}^{\text{ex}}}{k_{\text{B}}T}\right] \qquad \text{(S6)}$$

The same result was obtained by Sadowski et al.[2]

2 HCOOH DIMER AND MONOMER PARTIAL VAPOR PRESSURE

HCOOH monomer and dimer partial vapor pressures are calculated by[3]:

$$P_{M2}^{*} = (P_{M}^{*})^{2} \exp\left[\frac{2\mu_{\mathrm{M}}^{0} - \mu_{\mathrm{M2}}^{0}}{RT}\right] \quad \text{(S7)}$$

$$y_{\mathrm{M2}} = 1 - y_{\mathrm{M}} \quad \text{(S8)}$$

Combining Equations (S7) and (S8), a quadratic equation is obtained:

$$\frac{1 - y_{\mathrm{M}}}{y_{\mathrm{M}}^{2}} = \exp\left[\frac{2\mu_{\mathrm{M}}^{0} - \mu_{\mathrm{M2}}^{0}}{RT}\right] \cdot \frac{P}{P^{0}} \quad \text{(S9)}$$

where y_{M2} is HCOOH dimer vapor mole fraction, y_M is HCOOH monomer vapor mole fraction, R is the ideal gas constant, T is temperature, P is HCOOH partial vapor pressure, P^0 is the standard pressure (100 000 Pa) and μ_{M2}^{0} and μ_{M}^{0} are the excess chemical potentials of a dimer and monomer, equal to -716.59 kJ mol^{-1} and -351 kJ mol^{-1}, respectively[4]. All results for the HCOOH/H_2O system without addition of NaCl are shown in Table S12.

Table S1. Geometry for the HCOOH model optimized using Gaussian09[5] at the B3LYP/6-31G(d) level of theory.

**Atom**	***x*/[Å]**	***y*/[Å]**	***z*/[Å]**
C_fa1	0.133025	0.400057	-0.000002
O_fa1	-1.120802	-0.091884	0.000001
O_fa2	1.139287	-0.262302	0.000001
H_fa1	0.093278	1.498951	0.000004
H_fa2	-1.039312	-1.065806	0

Table S2. Interaction parameters for the formic acid FF-0 force field[6]. Lennard-Jones interactions between different atoms are computed using the Lorentz-Berthelot mixing rules[7]. It is important to note that there are exceptions/overrides to the use of the LB mixing rules. For some atom pairs, a minimum distance between two atoms (R_{min}) is specified.

**Atom**	**σ/[Å]**	**ε/k_B/[K]**	**q/[e^-]**
C_fa1	3.75	52.7999	0.52
O_fa1	3	85.51419	-0.53
O_fa2	2.96	105.72007	-0.44
H_fa1	2.42	7.57721	0
H_fa2	1	1	0.45
	**overrides**		
**Atom pair**	**σ/[Å]**	**ε/k_B/[K]**	**R_{min} /[Å]**
O_fa2 – H_fa2	1.98	10.28202651	1.4

Table S3. Interaction parameters for the formic acid FF-1 force field[6]. It is important to note that there are exceptions/overrides to the use of the LB mixing rules. For some atom pairs, a minimum distance between two atoms (R_{min}) is specified.

**Atom**	***σ*/[Å]**	ε/k_B/[K]	***q*/[e⁻]**
C_fa1	3.64	43.29832	0.5148
O_fa1	2.91	69.39759	-0.5247
O_fa2	2.87	86.47637	-0.4356
H_fa1	2.35	6.13393	0
H_fa2	1	1	0.4455
	**overrides**		
**Atom pair**	***σ*/[Å]**	ε/k_B/[K]	R_{min} /[Å]
O_fa2 – H_fa2	1.935	9.299	1.4

Table S4. Interaction parameters for the formic acid FF-2 force field[6], water SPC/E[8], CO_2[9] and NaCl[10] force fields. It is important to note that there are exceptions/overrides to the use of the LB mixing rules. For some atom pairs, a minimum distance between two atoms (R_{min}) is specified.

**Atom**	σ/[Å]	ε/k_B/[K]	q/[e$^-$]
C_fa1	3.67	49.6728	0.52
O_fa1	2.94	80.46271	-0.53
O_fa2	2.9	99.34559	-0.44
H_fa1	2.37	7.09611	0
H_fa2	1	1	0.45
O_SPCE	3.166	78.17706	-0.8476
H_SPCE	0	0	0.4238
O_CO2	3.017	85.671	-0.3256
C_CO2	2.742	29.93	0.6512
Na	2.159	177.457	1
Cl	4.83	6.434	-1

overrides

**Atom pair**	σ/[Å]	ε/k_B/[K]	R_{min} /[Å]
O_CO2 – O_SPCE	3.058	79.14	1.529
C_CO2 – O_SPCE	3.052	53.04	1.527
O_fa2 – H_fa2	1.95	9.967	1.4

Table S5. Interaction parameters for the formic acid FF-3 force field[11].

**Atom**	σ/[Å]	ε/k_B/[K]	q/[e^-]
C_fa1	3.2335	59.993	0
O_fa1	3.1496	85.053	-0.31574
O_fa2	2.9953	96.696	-0.42186
H_fa1	0	0	0.29364
H_fa2	0	0	0.44396

Table S6. Compositions and average box volumes of all 7 systems simulated for the Gibbs-Duhem integration test. The HCOOH FF-2 force field[6] and water SPC/E force field[8] were used.

x_{HCOOH}	N_{HCOOH}	N_{H2O}	V_{box}/[Å^3]
0	0	400	12053.37
0.1	40	360	13391.47
0.3	120	280	16324.03
0.5	200	200	19186.16
0.7	280	120	21981.36
0.9	360	40	24690.59
1	400	0	26031.60

Table S7. Compositions and average box volumes for all 28 systems simulated for the computation of Henry coefficients of CO_2. HCOOH pseudo-mole fractions were used, which are defined by: $x_{\mathrm{HCOOH}} = \frac{N_{\mathrm{HCOOH}}}{N_{\mathrm{HCOOH}}+N_{\mathrm{H_2O}}}$. For each HCOOH pseudo-mole fraction, four concentrations of NaCl are considered: 0, 0.25, 0.5, 0.75 mol NaCl per kilogram of solvent (H_2O + HCOOH).

x_{HCOOH}	N_{HCOOH}	N_{H2O}	N_{NaCl}	V_{box}/[Å^3]
0	0	400	0	12115.11
	0	400	2	12147.29
	0	400	4	12206.27
	0	400	5	12223.00
0.1	40	360	0	13471.09
	40	360	2	13513.98
	40	360	4	13582.70
	40	360	6	13633.51
0.3	120	280	0	16378.56
	120	280	3	16472.98
	120	280	5	16536.55
	120	280	8	16654.32
0.5	200	200	0	19271.93
	200	200	3	19371.17
	200	200	6	19467.67
	200	200	10	19605.28
0.7	280	120	0	22040.87
	280	120	4	22171.52
	280	120	8	22347.01
	280	120	11	22477.45
0.9	360	40	0	24752.86
	360	40	4	24916.40
	360	40	9	25141.52
	360	40	13	25345.05
1	400	0	0	26100.08
	400	0	5	26279.19
	400	0	9	26538.52
	400	0	14	26779.70

Table S8. Average values of the excess chemical potentials for HCOOH and H_2O obtained from (1) the probability distribution $p(\lambda)$, and (2) thermodynamic integration. The HCOOH FF-2 force field[6] and water SPC/E force field[8] were used. T = 298 K and P = 1 bar. The values are listed depending on HCOOH mole fraction in the system. The subscripts show uncertainties computed as the standard deviation. The values of the excess chemical potential are in units of kJ mol^{-1}.

**Compound**	**Route**	x_{HCOOH}						
		**0**	**0.1**	**0.3**	**0.5**	**0.7**	**0.9**	**1**
HCOOH	$p(\lambda)$	$-23.3_{0.3}$	$-24.4_{0.3}$	$-26.0_{0.3}$	$-26.1_{0.2}$	$-26.0_{0.2}$	$-25.6_{0.2}$	$-25.63_{0.08}$
	$\frac{\partial U}{\partial \lambda}$	$-23.2_{0.2}$	$-24.5_{0.1}$	$-26.1_{0.1}$	$-26.1_{0.1}$	$-25.9_{0.1}$	$-25.55_{0.04}$	$-25.4_{0.1}$
H_2O	$p(\lambda)$	$-29.7_{0.2}$	$-29.21_{0.08}$	$-28.4_{0.2}$	$-27.6_{0.2}$	$-27.3_{0.3}$	$-26.8_{0.2}$	$-26.7_{1.7}$
	$\frac{\partial U}{\partial \lambda}$	$-29.7_{0.1}$	$-29.30_{0.08}$	$-28.22_{0.09}$	$-27.2_{0.2}$	$-27.2_{0.1}$	$-27.0_{0.2}$	$-26.8_{0.3}$

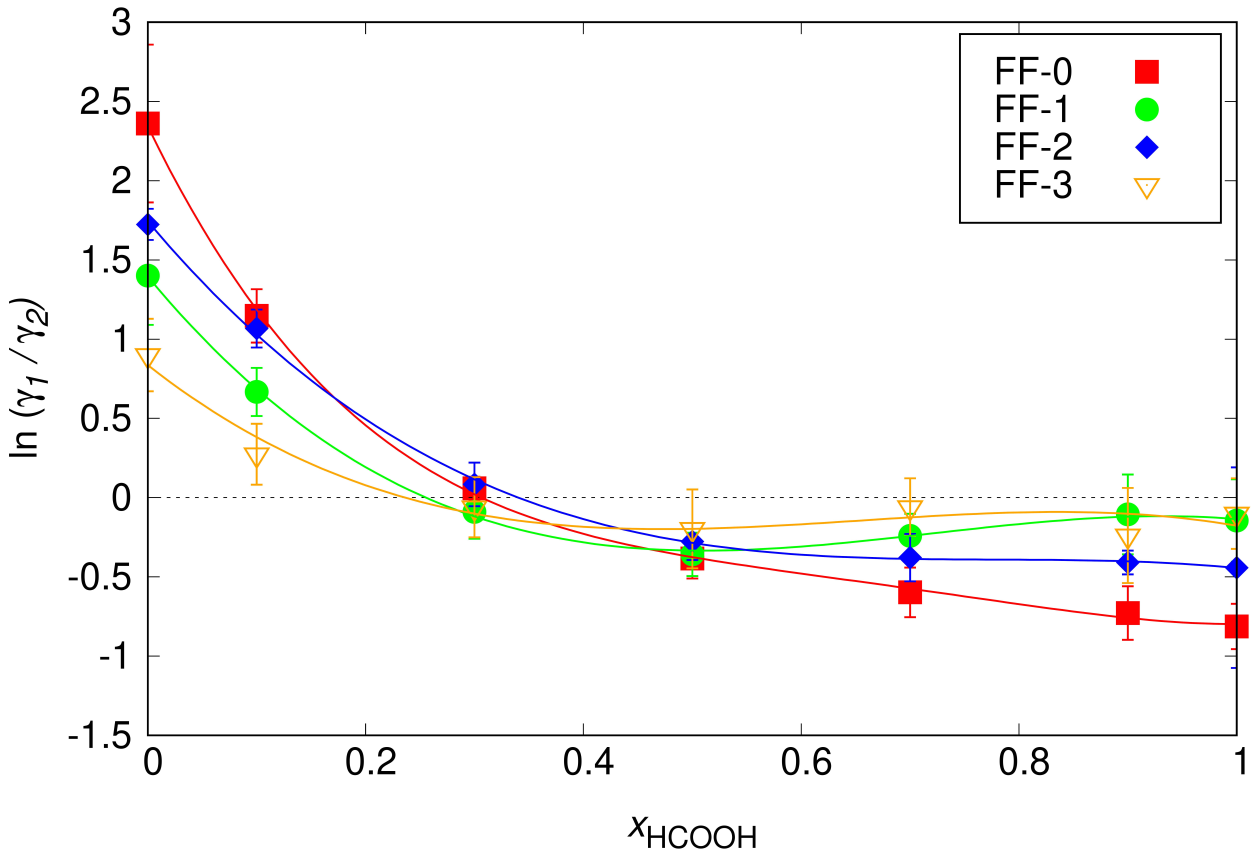

Figure S1. Gibbs-Duhem integration test for the four studied HCOOH/H_2O force fields FF-0[6], FF-1[6], FF-2[6] and FF-3[11] as a function of the mole fraction of HCOOH. The symbols represent data points for $\ln\left(\frac{\gamma_1}{\gamma_2}\right)$ obtained from the simulations for x_{HCOOH} = 0, 0.1, 0.3, 0.5, 0.7, 0.9, 1. The lines connecting the symbols are cubic polynomials (for FF-1, FF-2, FF-3) and quartic polynomial (for FF-0). The 101 data points for $\ln\left(\frac{\gamma_1}{\gamma_2}\right)$ in a range $x_{HCOOH} \in [0, 1]$ were obtained by interpolating the computed values with the use of polynomials. All the studied force fields resulted in the Gibbs-Duhem integral equals to zero within the error bars. The results for the Gibbs-Duhem integration test were as follows:

— FF-0: -0.08 ± 0.10,

— FF-1: -0.01 ± 0.13,

— FF-2: 0.03 ± 0.11,

— FF-3: -0.02 ± 0.16.

The uncertainties of the Gibbs-Duhem integrals were calculated using error propagation rules, see Eqs. 7–9 of the manuscript.

Table S9. Average values of activity coefficients for HCOOH and H_2O, computed based on the values of μ_{ex} of HCOOH and H_2O from the probability distribution of λ. The HCOOH FF-0, FF-1, FF-2 and FF-3 force fields[6,11] and water SPC/E force field[8] were used. T = 298 K and P = 1 bar. The listed values depend on the compound mole fraction in the system. The subscripts show uncertainties computed as the standard deviation obtained from 5 independent simulations. The activity coefficients at the limit $x_i = 0$ are calculated for 1 theoretical molecule of the compound i in the system.

	**FF-0**		**FF-1**		**FF-2**		**FF-3**	
x_i	γ_{HCOOH}	γ_{H2O}	γ_{HCOOH}	γ_{H2O}	γ_{HCOOH}	γ_{H2O}	γ_{HCOOH}	γ_{H2O}
0	$11.9_{5.7}$	$2.3_{0.3}$	$4.3_{1.3}$	$1.2_{0.4}$	$5.6_{0.5}$	$1.8_{0.9}$	$2.3_{0.6}$	$1.1_{0.2}$
0.1	$3.6_{0.3}$	$1.9_{0.2}$	$2.1_{0.3}$	$1.1_{0.2}$	$3.2_{0.5}$	$1.6_{0.1}$	$1.3_{0.1}$	$1.2_{0.1}$
0.3	$1.4_{0.2}$	$1.8_{0.2}$	$1.1_{0.2}$	$1.3_{0.2}$	$1.4_{0.1}$	$1.5_{0.2}$	$1.1_{0.2}$	$1.1_{0.1}$
0.5	$1.2_{0.1}$	$1.71_{0.05}$	$0.9_{0.1}$	$1.3_{0.1}$	$1.1_{0.1}$	$1.5_{0.2}$	$0.9_{0.1}$	$1.2_{0.1}$
0.7	$1.0_{0.1}$	$1.3_{0.1}$	$1.01_{0.09}$	$1.2_{0.1}$	$1.01_{0.04}$	$1.3_{0.2}$	$1.1_{0.1}$	$1.1_{0.1}$
0.9	$0.9_{0.1}$	$1.11_{0.09}$	$1.00_{0.09}$	$1.1_{0.1}$	$1.1_{0.1}$	$1.11_{0.07}$	$1.0_{0.3}$	$1.0_{0.1}$
1	1	1	1	1	1	1	1	1

Table S10. Average values of densities in units of mol m^{-3} for HCOOH and H_2O, computed from the probability distribution of λ. The HCOOH FF-0, FF-1, FF-2 and FF-3 force fields[6,11] and water SPC/E force field[8] were used. T = 298 K and P = 1 bar. The listed values depend on the compound mole fraction in the system. The subscripts show uncertainties computed as the standard deviation obtained from 5 independent simulations. The densities at the limit x_i = 0 are calculated for 1 theoretical molecule of the compound i in the system.

	**FF-0**		**FF-1**		**FF-2**		**FF-3**	
x_i	ρ_{HCOOH}	ρ_{H2O}	ρ_{HCOOH}	ρ_{H2O}	ρ_{HCOOH}	ρ_{H2O}	ρ_{HCOOH}	ρ_{H2O}
0[a]	$137.9_{0.2}$	$61.47_{0.01}$	$137.8_{0.1}$	$62.97_{0.04}$	$137.7_{0.2}$	$63.78_{0.02}$	$137.7_{0.2}$	$72.7_{0.1}$
0.1	$4919.3_{9.8}$	$2595.8_{1.8}$	$4959.8_{8.1}$	$2660.8_{3.4}$	$4961.2_{8.2}$	$2690.4_{2.5}$	$5067.2_{7.7}$	$3045.5_{2.1}$
0.3	$11998.1_{7.7}$	$8785.9_{10.3}$	$12185.0_{19.0}$	$8990.8_{5.3}$	$12209.9_{20.4}$	$9066.3_{3.1}$	$13034.3_{16.5}$	$10142.9_{4.5}$
0.5	$16877.9_{17.2}$	$16876.2_{17.2}$	$17196.5_{27.9}$	$17194.8_{27.9}$	$17313.8_{16.2}$	$17312.1_{16.2}$	$19003.7_{10.6}$	$19001.8_{10.6}$
0.7	$20502.5_{23.9}$	$27992.7_{18.0}$	$20980.5_{12.4}$	$28428.9_{44.4}$	$21156.7_{7.1}$	$28486.9_{47.7}$	$23669.2_{10.5}$	$30410.3_{38.5}$
0.9	$23364.6_{15.8}$	$44269.3_{88.2}$	$23949.6_{30.8}$	$44633.8_{73.1}$	$24216.5_{22.5}$	$44646.8_{73.4}$	$27412.0_{19.0}$	$45600.3_{69.2}$
1	$24595.9_{5.5}$	$55157.8_{63.1}$	$25196.7_{16.4}$	$55147.5_{58.2}$	$25520.7_{9.8}$	$55114.5_{91.0}$	$29086.5_{22.2}$	$55091.9_{60.5}$

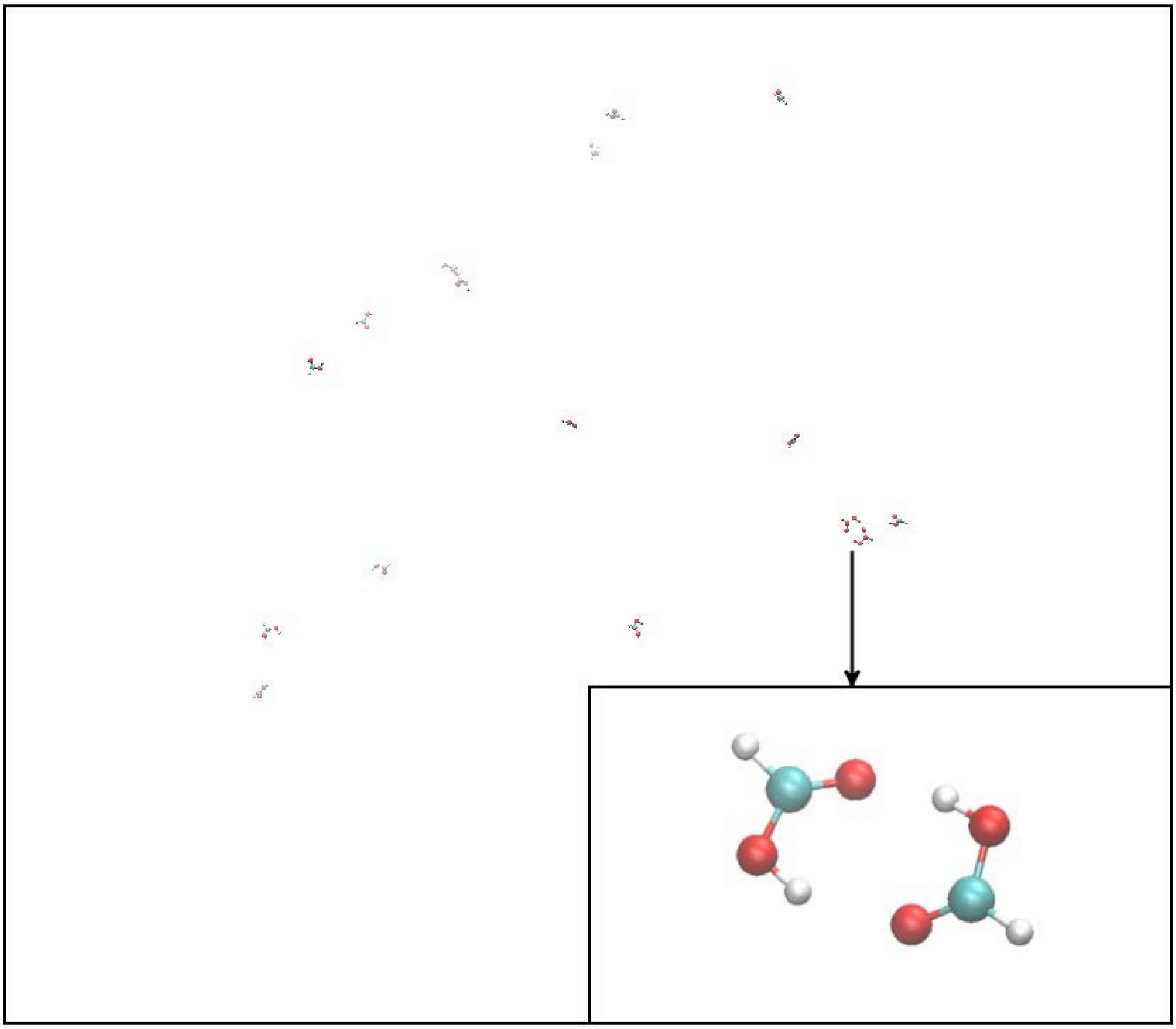

Figure S2. Visualization of a pure HCOOH system configuration in the gas phase at $T = 360$ K, $P = 0.7$ bar, using the VMD software[12]. The HCOOH FF-2 force field[6] was used. The presence of dimers was confirmed. A typical example of a dimer is highlighted in red, and its enlarged image is shown in the lower right corner.

Table S11. Comparison of the saturated vapor pressures of pure HCOOH (computed from series of *NPT* simulations of the vapor phase and calculated using the liquid phase properties from Gibbs Ensemble simulations) with experimental values[11], as a function of temperature. The HCOOH FF-2 force field[6] and water SPC/E force field[8] were used. The subscripts show uncertainties computed as the standard deviation obtained from five independent Gibbs Ensemble simulations. The uncertainties of the pure HCOOH vapor pressures computed from the series of *NPT* simulations ($P_{\mathrm{HCOOH,sim}}$) and calculated using Eq. 10, are close to zero due to the small number of molecules in the vapor phase.

T/[K]	$P_{\mathrm{HCOOH,sim}}$/[MPa]	$P_{\mathrm{HCOOH,calc}}$/[MPa]	$P_{\mathrm{HCOOH,exp}}$/[MPa]
335	$0.019_{0.003}$	$0.037_{0.022}$	0.027
360	$0.057_{0.013}$	$0.077_{0.03}$	0.066
385	$0.220_{0.02}$	$0.176_{0.031}$	0.140
410	$0.443_{0.024}$	$0.343_{0.008}$	0.269
435	$0.694_{0.09}$	$0.573_{0.013}$	0.480
460	$1.232_{0.124}$	$0.947_{0.02}$	0.803
485	$1.720_{0.06}$	$1.431_{0.014}$	1.275
510	$2.819_{0.313}$	$2.086_{0.01}$	1.937
535	$3.381_{0.205}$	$2.922_{0.023}$	2.839
560	$4.872_{0.189}$	$3.988_{0.036}$	4.036

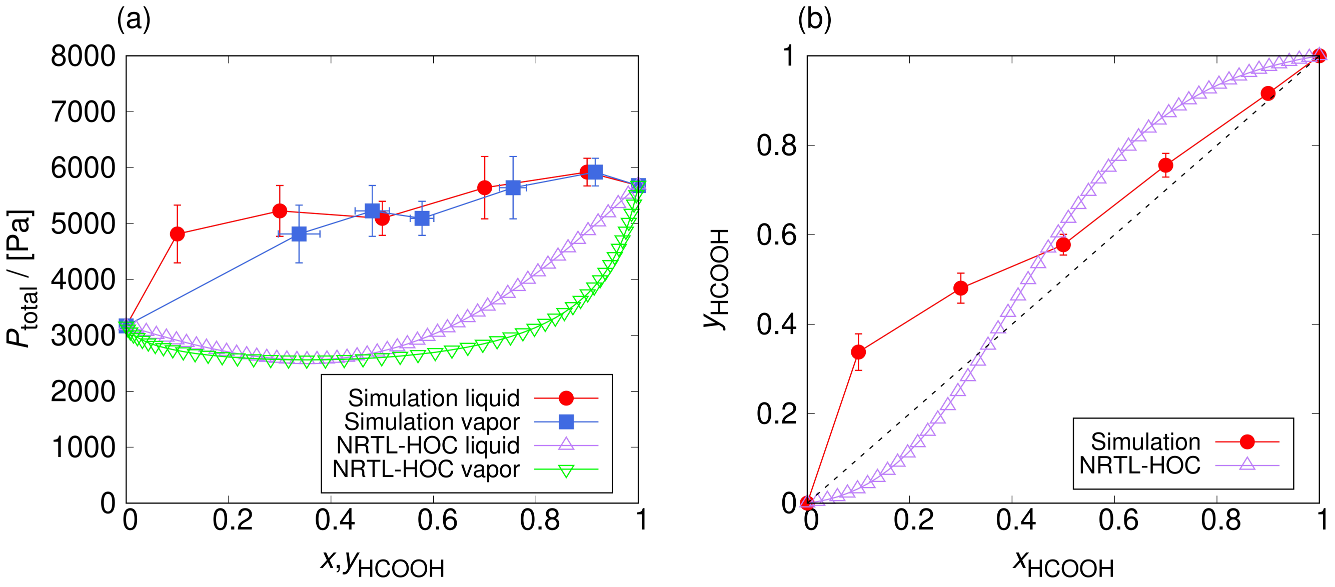

Figure S3. The azeotropic behavior of the HCOOH/H_2O system without addition of NaCl: (a) total vapor pressure ($P_{H2O} + P_{HCOOH}$) as a function of the mole fractions of HCOOH in the vapor and liquid, (b) mole fraction of HCOOH in the liquid phase as a function of vapor mole fraction. The lines connecting the symbols are used to guide the eye. The simulated HCOOH/H_2O systems show a low-boiling azeotrope behavior in sharp contrast to the high-boiling azeotrope obtained from NRTL-HOC calculations[13]. The uncertainties were computed as the standard deviation from five independent simulations.

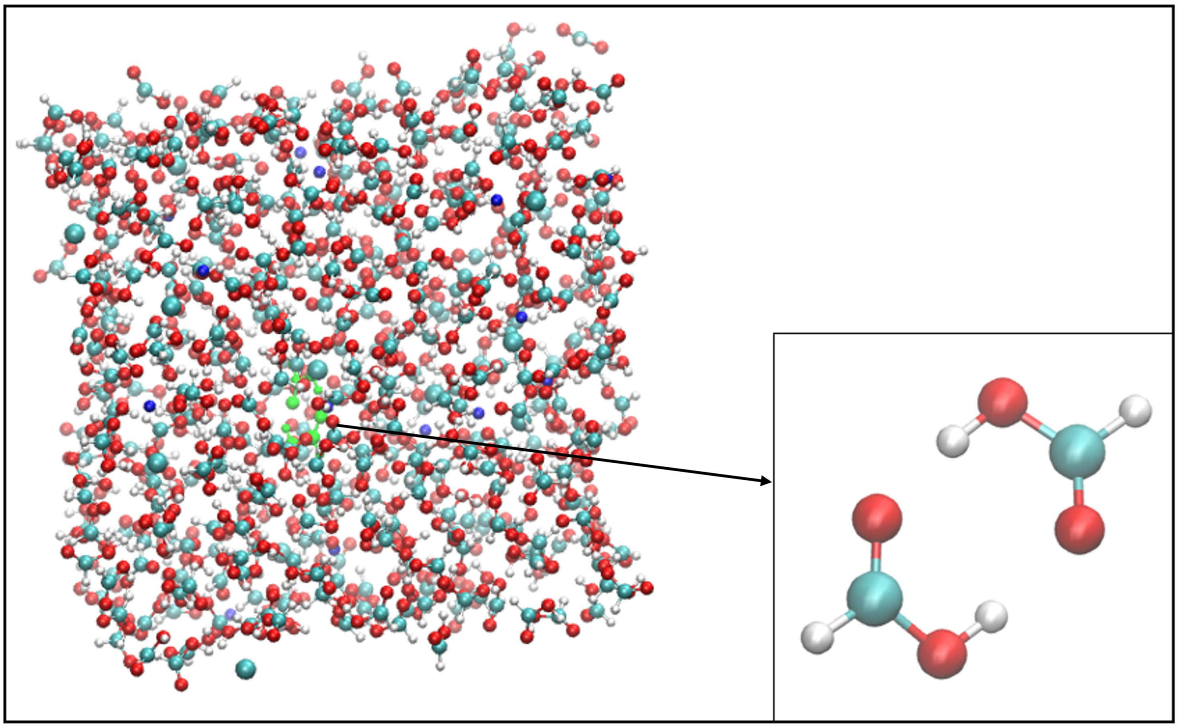

Figure S4. Visualization of a $HCOOH/H_2O/CO_2/NaCl$ system configuration at T = 298 K, P = 1 bar, using the VMD software[12]. The pseudo-mole fraction of HCOOH in the system was x_{HCOOH} = 1 and the concentration of NaCl added was 0.75 mol NaCl per kilogram of solvent (HCOOH + H_2O). The HCOOH FF-2 force field[6], water SPC/E force field[8], CO_2 force field[9] and NaCl[10] force field were used. The presence of dimers was confirmed in the gas phase. A typical example of a dimer is highlighted in green, and its enlarged image is shown in the lower right corner.

Table S12. The HCOOH dimer and monomer partial vapor pressures calculated for the HCOOH/H_2O system without addition of NaCl. The HCOOH FF-2 force field[6], water SPC/E force field[8] and CO_2 force field[9] were used. The subscripts show uncertainties computed using error propagation rules. The calculated dimer partial vapor pressures are found to be approximately 2 – 4 times higher than monomer partial vapor pressures, confirming that the non-ideal dimer formation behavior is the reason, why the studied model does not reproduce vapor pressures and azeotropic behavior more precisely than the order of magnitude.

x_{HCOOH}	P_{HCOOH}/[Pa]	P^*_{M2}/[Pa]	P^*_{M}/[Pa]
0.1	$1615.7_{196.3}$	$1070.9_{8.9}$	$544.8_{8.9}$
0.3	$2510.4_{304.8}$	$1803.5_{11.6}$	$706.9_{11.6}$
0.5	$2946.6_{284.6}$	$2171.0_{8.1}$	$775.6_{8.1}$
0.7	$4271.5_{543.6}$	$3313.3_{17.3}$	$958.2_{17.3}$
0.9	$5422.1_{236.5}$	$4327.1_{2.3}$	$1095.0_{2.3}$
1	5678.2	4554.7	1123.5

REFERENCES

(1) Ahmadreza Rahbari. *Thermodynamics of Industrially Relevant Systems: Method Development and Applications*; PhD thesis, Delft University of Technology, Delft, The Netherlands, 2020. DOI:10.4233/uuid:eb04d860-281a-4c6b-8c5b-263f526d0bd9

(2) Hempel, S.; Fischer, J.; Paschek, D.; Sadowski, G. Activity Coefficients of Complex Molecules by Molecular Simulation and Gibbs-Duhem Integration. *Soft Materials* **2012**, *10*, 26-41.

(3) McQuarrie, D. A.; Simon, J. D., *Physical chemistry: a molecular approach*, 1st ed. University Science Books: Sausalito, California, 1997.

(4) Chao, J.; Zwolinski, B. J. Ideal Gas Thermodynamic Properties of Methanoic and Ethanoic Acids. *Journal of Physical and Chemical Reference Data* **1978**, *7*, 363-377.

(5) Frisch, M. J.; Trucks, G. W.; Schlegel, H. B.; Scuseria, G. E.; Robb, M. A.; Cheeseman, J. R.; Scalmani, G.; Barone, V.; Mennucci, B.; Petersson, G. A., et al. *Gaussian 09*, Revision A.1; Gaussian, Inc.: Wallingford, CT, 2009.

(6) Salas, F. J.; Núñez-Rojas, E.; Alejandre, J. Stability of Formic acid/pyridine and Isonicotinamide/formamide Cocrystals by Molecular Dynamics Simulations. *Theoretical Chemistry Accounts* **2017**, *136*, 17.

(7) Allen, M. P.; Tildesley, D. J. *Computer simulation of liquids (2nd ed.)*. Oxford, United Kingdom: Oxford University Press, 2017.

(8) Berendsen H. J.; Grigera J. R.; Straatsma T. P. The Missing Term in Effective Pair Potentials. *The Journal of Physical Chemistry* **1987**, *91*, 6269-6271.

(9) García-Sánchez, A.; Ania, C. O.; Parra, J. B.; Dubbeldam, D.; Vlugt, T. J. H.; Krishna, R.; Calero, S. Transferable Force Field for Carbon Dioxide Adsorption in Zeolites. *The Journal of Physical Chemistry C* **2009**, *113*, 8814-8820.

(10) Aragones, J. L.; Sanz, E.; Vega, C. Solubility of NaCl in Water by Molecular Simulation Revisited. *The Journal of Chemical Physics 2012*, *136*, 244508.

(11) Schnabel, T.; Cortada, M.; Vrabec, J.; Lago, S.; Hasse, H. Molecular Model for Formic Acid Adjusted to Vapor–liquid Equilibria. *Chemical Physics Letters* **2007**, *435*, 268-272.

(12) Humphrey, W.; Dalke, A.; Schulten, K. VMD - Visual Molecular Dynamics. *J. Molec. Graphics* **1996**, *14*, 33-38.

(13) Ramdin, M.; Morrison, A. R. T.; de Groen, M.; van Haperen, R.; de Kler, R.; Irtem, E.; Laitinen, A. T.; van den Broeke, L. J. P.; Breugelmans, T.; Trusler, J. P. M.; et al. High-Pressure Electrochemical Reduction of CO_2 to Formic Acid/Formate: Effect of pH on the Downstream Separation Process and Economics. *Industrial & Engineering Chemistry Research* **2019**, *58*, 22718-22740.
